# Endoplasmic Reticulum Stress in Colonic Mucosa of Ulcerative Colitis Patients Is Mediated by PERK and IRE1 Pathway Activation

**DOI:** 10.1155/2022/6049500

**Published:** 2022-02-09

**Authors:** Bruno Lima Rodrigues, Isabella Dotti, Lívia Bitencourt Pascoal, Joseane Morari, Miriam Esteller, Andressa Coope, Maria de Lourdes Setsuko Ayrizono, Azucena Salas, Raquel Franco Leal

**Affiliations:** ^1^Inflammatory Bowel Disease Research Laboratory, Gastrocenter, Colorectal Surgery Unit, School of Medical Sciences, University of Campinas (UNICAMP), Campinas, Brazil; ^2^Department of Gastroenterology, IDIBAPS, Hospital Clínic, CIBER-EHD, Barcelona, Spain; ^3^Laboratory of Cell Signaling, Obesity and Comorbidities Research Center, School of Medical Sciences, University of Campinas (UNICAMP), Campinas, Brazil

## Abstract

Ulcerative colitis (UC) is characterized by a chronic overproduction of proinflammatory cytokines. During an acute phase, the endoplasmic reticulum (ER) is overloaded and the protein folding process is impaired, a condition named ER stress. This state induces a response (unfolded protein response (UPR)), initiated by the activation of IRE1/Xbp-1, PERK/eIF2*α*, and ATF6 pathways, which has previously been linked to intestinal inflammation in experimental models. ER stress and UPR activation trigger the activation of proinflammatory, autophagy, and apoptosis genes, in addition to promoting protein degradation. Therefore, the goal of this study was to evaluate the activation of ER stress and UPR in colonic mucosa of UC patients. *Patient and Methods*. Transcriptional analysis of ER stress- and UPR-related genes was performed by qPCR from intestinal mucosa of patients with UC. We also performed in situ hybridization (ISH) and immunohistochemistry (IHQ) of PERK/eIF2*α* and IRE1/Xbp-1 pathways and UPR-related chaperones. *Results*. We first evaluated inflammatory genes via qPCR, and we observed that all analyzed proinflammatory transcripts were upregulated in UC patients. ISH and IHQ images showed that ER stress is activated via PERK/eIF2*α* and IRE1/Xbp-1 pathways not only in intestinal epithelial cells but also in cells of the lamina propria of UC colonic mucosa. Transcriptional analysis confirmed that EIF2AK3 was upregulated in UC patients. UPR-related genes, such as ATF3, STC2, and DDIT3, along with the chaperones and cochaperones DNAJC3, CALR, HSP90B1, and HSPA5, were also upregulated in UC patients. In addition, we observed that proapoptotic and autophagy genes (Bax and ATG6L1, respectively) were also upregulated. *Conclusion*. Our results suggest that ER stress and UPR are indeed activated in UC patients and this may contribute to the chronic inflammatory process seen in UC. The increased apoptosis and autophagy markers further support the activation of these findings once they are activated to counterbalance tissue damage. These findings provide new insights into the molecular mechanisms that maintain UC activity and open new possibilities to attenuate intestinal inflammation.

## 1. Introduction

Ulcerative colitis (UC) refers to a chronic inflammatory bowel disease (IBD) with a relapsing and remitting course that affects the rectum and extends to proximal segments of the colon. It is characterized by abdominal pain, faecal urgency, and diarrhea [1]. In the last decade, UC became a public health issue worldwide once the annual direct and indirect costs rose above US$10 billion in the USA and Є15 billion in Europe [2]. Although the pathogenesis of UC involves genetic aspects, the immune response plays a relevant role on the onset and maintenance of the disease [3].

The increase in the production of proinflammatory cytokines is one of the many reasons that overloads the endoplasmic reticulum (ER), impairing the folding of proteins and their final conformation. The accumulation of unfolded proteins in the ER lumen leads to a condition named endoplasmic reticulum stress (ER stress) [4].

In order for the cells to prevent the cytotoxicity promoted by ER stress, the unfolded protein response (UPR) is activated [5]. In eukaryotic cells, the UPR signalling pathway is mainly mediated by three transmembrane proteins: inositol-requiring transmembrane kinase/endonuclease 1 (IRE1), protein kinase-like endoplasmic reticulum kinase (PERK), and activating transcription factor 6 (ATF6) [6].

Once UPR is activated, mRNA translation is moderated through the phosphorylation of eIF2*α* [7]. The phosphorylated eIF2*α* (p-eIF2*α*) selectively enhances the translation of activating transcription factor 4 (ATF4) that is involved in protein folding and ER stress-induced apoptosis [8]. IRE1 is presented in two isoforms: IRE*α* and IRE*β*, both of them undergo dimerization and autophosphorylation after activation. IRE1*α* is able to decrease the load of newly synthesized proteins in ER lumen through the degradation of mRNA [9]. In addition, once activated, IRE1's endoribonuclease and kinase activity promotes the activation of Jun-related kinase (JNK) and NF-*κ*B [10, 11] and splicing of X-box binding protein-1 (Xbp1), which regulates the expression of critical genes related to chaperone production and ER-associated protein degradation (ERAD) components [12]. ATF6, when activated, undergoes proteolytic cleavage in the Golgi apparatus. Then, its released fragments are translocated to the nucleus in order to activate the transcription of a limited number of genes with ER quality control functions, many of which are already induced by Xpb-1 [13, 14].

Studies using Xbp-1 gene and IRE1*β* knockout mice showed increased intestinal inflammation, as well as an impaired intestinal barrier, which led to the increase in pathogen invasion [10, 15]. The PERK pathway is indirectly related to IBD via DDIT3 activation (DNA damage inducible transcript 3, also known as CHOP/GADD153), responsible for ER stress-related apoptosis [10, 16]. ATF6 knockout mice have shown a decrease in the expression of ER chaperone-related genes [17]. Furthermore, a recent study by our research group has shown that EIF2AK3 and ERN1 genes were upregulated in the intestinal mucosa of patients with Crohn's disease, another form of IBD [18].

Therefore, the goal of this study was to characterize ER stress activation in UC patients in a multicentre study using transcriptional analysis and in situ hybridization of UPR-related genes, as well as to determine the presence of UPR-related proteins using immunohistochemistry technique. The identification of the activated ER stress pathways in UC may contribute to better understanding of its physiopathology and to the development of therapeutic targets which can be explored in the future.

## 2. Material and Methods

### 2.1. Patients and Ethics

The study was conducted in accordance with the Declaration of Helsinki, and it was approved by the Ethics Committee of the University of Campinas and by the Ethics Committee of the Hospital Clinic. All patients provided a written informed consent form for study participation.

The UC group was composed of samples from affected intestinal mucosa taken from patients who presented active UC during colonoscopy. The control group was composed of samples from normal mucosa taken from patients who underwent colonoscopy for noninflammatory disease.  Table 1 shows demographic and clinical data from patients included in the study. UC activity was confirmed by Mayo score. Only patients who presented Mayo endoscopic score > 1 were included in the study. UC patients who presented normal mucosa (i.e., without erythema and without erosions and/or ulcers), or the ones who presented a Mayo endoscopic score < 1, were excluded from the study.

The study was carried out at the IBD Research Laboratory, from the School of Medical Sciences of University of Campinas (UNICAMP), Brazil, and at the Gastroenterology Laboratory, from the August Pi I Sunyer Biomedical Research Institute (IDIBAPS), located in Barcelona, Spain.

### 2.2. RNA Extraction and cDNA Synthesis

Total RNA was extracted using the RNeasy Mini Kit (Qiagen, Cat No./ID: 74104), according to the manufacturer's instructions. The concentration and purity of the extracted RNA were determined using UV spectrophotometry at 260 nm. cDNA synthesis was carried out using High-Capacity cDNA Reverse Transcription Kit (Applied Biosystems, Foster, City, CA, USA).

### 2.3. Quantitative Real-Time PCR (qPCR)

Real-time quantitative PCR reactions were performed using TaqMan™ system (Applied Biosystems). To evaluate the activation of the three main branches from UPR, the primers used were EIF2AK3 (Hs_00178128_m1), ERN1 (Hs_00980095_m1), and ATF6*α* (Hs_00232586_m1). To evaluate the inflammatory profile, as well as apoptotic and autophagy genes, the primers used were IL-6 (Hs00174131_m1), IL-10 (Hs00961622_m1), IFN-*γ* (Hs_00989291_m1), IL-1*β* (Hs_01555410_m1), IL-23p19 (Hs_00372324_m1), TNF-*α* (Hs_00174128_m1), IL-17 (Hs_00174383_m1), IL22 (Hs01574154_m1), Bcl-2 (Hs_00608023_m1), Bax (Hs_00180269_m1), and ATG16L1 (Hs_00250520_m1). To evaluate UPR-related genes and chaperones, the primers used were ATF3 (Hs_00231069_m1), CALR (Hs_00189032_m1), STC2 (Hs_00175027_m1), DNAJC3 (Hs_00534483_m1), DDIT3 (Hs_0109850_m1), HSP90B1 (Hs_00427665_g1), and HSPA5 (Hs_99999174_m1). The transcriptional levels of the target genes were normalized using GAPDH as the endogenous control gene. Gene expression was then determined using fold change, obtained by delta-delta Ct method.

### 2.4. Hematoxylin and Eosin (H&E)

Formalin-fixed and paraffin-embedded (FFPE) intestinal mucosa samples were cut at 4 *μ*m and stained with hematoxylin and eosin dye. Photomicrographs were taken using a DFC345FX (Leica, Germany) with control software (LAS V4.12, Leica). For the quantitative analysis, three random panchromatic objective fields of higher magnification (40x) of each sample were scanned and analyzed by two blinded observers (B.L.R. and R.F.L.).

### 2.5. Immunohistochemistry (IHC)

Formalin-fixed and paraffin-embedded (FFPE) intestinal mucosa samples were cut at 4 *μ*m. After deparaffinization, antigen retrieval was performed using a citrate buffer solution (pH 6.0) for 20 minutes at 95°C. Endogenous peroxidase was blocked with a hydrogen peroxide solution (3% H_2_O_2_ 10 vol.) followed by washes in phosphate-buffered saline (PBS, 10 mM, pH 7.4). Primary antibodies were diluted in a 1% bovine serum albumin (BSA) solution (diluted in PBS) and incubated 4°C overnight. The antibodies used were anti-phosphor-[Ser51] eIF2a (Abcam, ab32157, rabbit monoclonal) diluted 1/50 in BSA, purified anti-Xbp-1 (COOH terminus) (BioLegend, 555483, rabbit polyclonal) diluted 1/50 in BSA, anti-GRP78 (Abcam, ab21685, rabbit polyclonal) used in the concentration of 1 *μ*g/ml, anti-GRP94 (Santa Cruz Biotechnology, sc11402, rabbit polyclonal) diluted 1/50 in BSA, and anti-DDIT3 (BioVision, A1674-100), diluted 1/100 in BSA. Signal detection was determined with the use of an immunoperoxidase detection system (Vector Laboratories) and then incubated with DAB solution (Dako). The slides were then counterstained with hematoxylin, dehydrated with several alcohol concentrations, and mounted with Mounting Medium (Dako). Photomicrographs were taken using the DFC345FX (Leica, Germany) with control software (LAS V4.12, Leica) or Zeiss Axioplan 2 microscope with digital camera (Olympus DP–72) with control software (Cellsens). For the placement of scale bars in the figures, calibration was performed to guarantee that the structures were accurately measured, through the association of the number of pixels with the cell nucleus (10 *μ*m). All calibration and placement of bars were performed using the ImageJ program. For the quantitative analysis, three random panchromatic objective fields of higher magnification (40x) of each sample were scanned and analyzed by two blinded observers (B.L.R. and R.F.L.).

### 2.6. In Situ Hybridization (ISH)

In situ hybridization (ISH) was performed using the chromogenic RNAscope® 2.5 HD-RED Assay and the HybEZ™ Hybridization System (Advanced Cell Diagnostics, Hayward, CA, USA) according to the supplier's instructions. FFPE mucosa samples were cut at 5 *μ*m and deparaffinized through xylol and ethanol washes in absolute concentrations. Tissues were then treated with RNAscope Hydrogen Peroxide solution for 10 min at room temperature, RNAscope Target Retrieval Reagents solution for 15 min at >99°C in a steamer, and RNAscope Protease Plus for 30 min at 40°C. Tissues were then hybridized with ERN1 (497331) and EIF2aK3 (541471) mRNA probes for 2 h at 40°C. After rinsing with wash buffer, amplification of the hybridized probe signal was obtained by the serial application of Amp 1 to Amp 6. Fast Red solution was then applied for 10 minutes to detect the target RNAs. Sections were counterstained with hematoxylin and mounted with EcoMount (Thermo Fisher Scientific Inc., Waltham, MA). The ERN1 and EIF2aK3 mRNA signal was evaluated by the presence of punctuate dots using an Olympus BX51 microscope (Tokyo, Japan) and CellF Software.

### 2.7. Statistical Analysis

All data were analyzed and reported using median values. To assess normal distribution, the Kolmogorov-Smirnov test was conducted. Then, nonparametric Mann–Whitney test was performed between groups. A *p* value less than 0.05 was considered significant.

## 3. Results

### 3.1. Proinflammatory Cytokine Profile in the Colonic Mucosa of Ulcerative Colitis Patients

**Figure 1:**
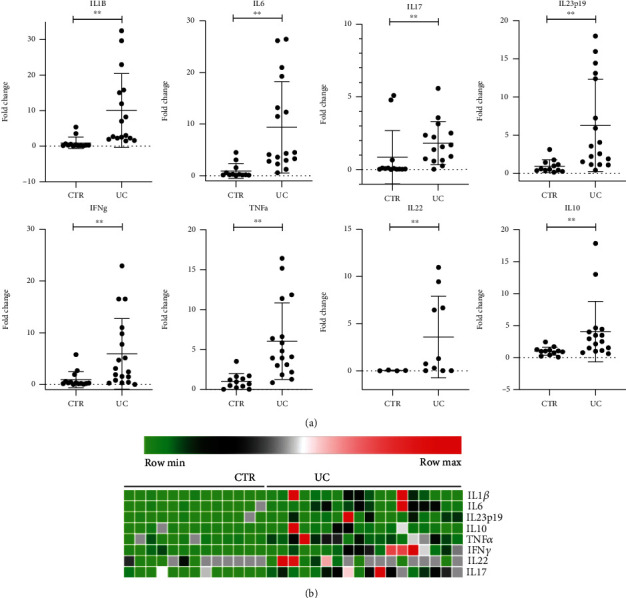
Transcriptional analysis of pro- and anti-inflammatory cytokines in UC patients. The transcriptional levels of the cytokines were determined by rt-PCR. (a) Transcriptional levels of IL1*β*, IL6, IL17, IL22, IL23p19, IFN*γ*, TNF*α*, and IL10 are shown as a fold change. (b) Heat map of the inflammatory cytokines. ^∗∗^*p* < 0.005. CTR = control; UC = ulcerative colitis.

Firstly, to confirm the proinflammatory signalling in the UC colonic mucosa, we evaluated the expression of cytokines often modulated in IBD patients by rt-PCR. For this study, we included 14 controls and 18 UC patients. The UC group presented a significant upregulation of the following proinflammatory cytokine transcripts: IL1*β*, IL6, IL17, IL22, IL23p19, IFN*γ*, and TNF*α* when compared to the control group (*p* < 0.005). The anti-inflammatory cytokine IL-10 was also significantly modulated (*p* < 0.005), showing a counteraction against the pronounced proinflammatory status of the colonic mucosa affected by UC (Figure 1(a)).  Figure 1(b) shows a heat map of the inflammatory cytokines in both groups. Therefore, type 1 (Th1) and 17 (Th17) immune responses, as well as innate response, are involved in the intestinal inflammation in UC. Supplementary figure (available here) shows representative hematoxylin and eosin (H&E) staining of intestinal mucosa to illustrate the cell infiltrate of the lamina propria in the UC and CTR groups.

### 3.2. PERK/eIF2*α* and IRE1/sXBP-1 but Not ATF6 Pathway Are Activated in the Colonic Mucosa of Ulcerative Colitis Patients

We chose to investigate which ER stress pathways and UPR-related genes were activated in UC patients. For this analysis, we included 14 controls and 18 UC patients for the rt-PCR, 5 controls and 8 UC patients for the immunohistochemistry analysis, and 3 controls and 4 UC patients for the in situ hybridization.

**Figure 2:**
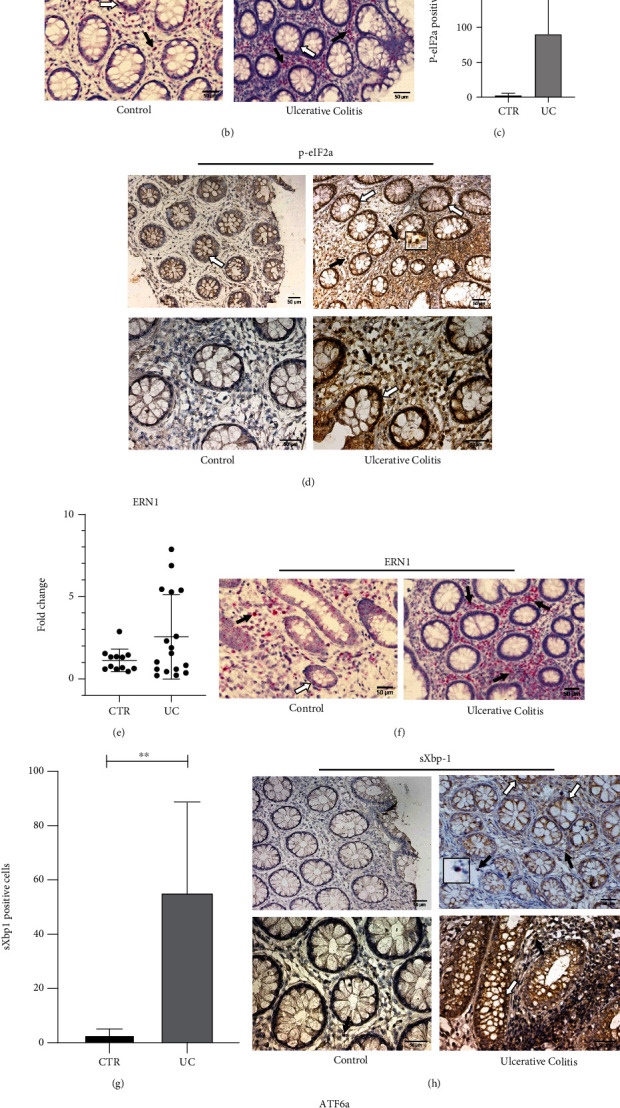
Activation of ER stress pathways in the colonic mucosa of UC patients. (a) Expression analysis of EIF2AK3 was performed by qPCR. Transcriptional levels of EIF2AK3 are shown as a fold change, ^∗^*p* < 0.05. (b) EIF2AK3 in situ hybridization was performed on FFPE slides from intestinal mucosa of the UC and CTR groups. The arrows indicate EIF2AK3-positive cells, which are shown as pink dots. The white arrows indicate positive epithelial cells and the black arrows signalize positive cells from the lamina propria. Scale bars: 50 *μ*m. (c) Quantitative analysis of immunohistochemistry staining for p-eIF2*α* of both groups. For UC, *n* = 5; for CTR, *n* = 6; ^∗∗^*p* < 0.005. (d) Immunohistochemical analysis of p-eIF2*α* was performed on paraffin-embedded slides from intestinal mucosa of both groups. The arrows indicate p-eIF2*α*-positive cells, which are shown in brown. The white arrows indicate positive epithelial cells, and the black arrows signalize positive cells from the lamina propria. Scale bars: 50 *μ*m. (e) Expression analysis of ERN1 was performed by qPCR. Transcriptional levels of ERN1 are shown as a fold change, *p* > 0.05. (f) ERN1 in situ hybridization was performed on FFPE slides from intestinal mucosa of both groups. The arrows indicate ERN1-positive cells, which are shown as pink dots. The white arrows indicate positive epithelial cells, and the black arrows signalize positive cells from the lamina propria. Scale bars: 50 *μ*m. (g) Quantitative analysis of immunohistochemistry staining for sXbp-1 of both groups. For UC, *n* = 4; for CTR, *n* = 6; ^∗∗^*p* < 0.005. (h) Immunohistochemical analysis for sXbp-1 was performed on FFPE slides from intestinal mucosa of both groups. The arrows indicate sXbp-1-positive cells, which are shown in brown. The white arrows indicate positive epithelial cells, and the black arrows signalize positive cells from the lamina propria. Scale bars: 50 *μ*m. (i) ATF6*α* transcriptional analysis was performed by rt-PCR. Transcriptional levels of ATF6 are shown as fold change, *p* > 0.05. CTR = control; UC = ulcerative colitis.

The first UPR pathway evaluated was the PERK/eIF2*α*. We performed rt-PCR technique to investigate the expression of EIF2AK3 gene, responsible for encoding PERK protein. As a result, we observed a significant upregulation (*p* < 0.05) in UC patients when compared with that in the control group (Figure 2(a)).

Additionally, by using in situ hybridization, we investigated the location of EIF2AK3 mRNA as well as, by immunohistochemistry, the protein expression of p-eIF2*α*. The transcriptional expression of EIF2AK3-positive cells was seen in the epithelial layer and in the lamina propria of control patients, and a marked increase of EIF2AK3 in the lamina propria cells of UC patients was observed (Figure 2(b)). Immunostaining revealed p-eIF2*α* expression in the epithelial layer and in the cells of the lamina propria, with more intense immunoreactivity in UC patients when compared to the controls (*p* < 0.05) (Figures 2(c) and 2(d)).

The second UPR branch evaluated was the IRE1/Xbp-1 pathway. There was no statistical difference in the transcriptional levels of ERN1 between the control and UC groups as determined by qPCR (*p* > 0.05) (Figure 2(e)). Nevertheless, the ISH revealed that ERN1 was upregulated in the lamina propria, of the UC samples (Figure 2(f)). Since Xbp-1 is a protein expressed upon IRE1 activation, its evaluation is considered an accurate form to confirm whether IRE1 has been indeed activated or not. Therefore, we performed immunohistochemistry analysis using a specific antibody that recognizes the spliced variant of Xbp-1 protein (sXbp-1) to investigate protein expression of sXbp-1. Then, we observed significantly higher expression of sXbp-1, mainly in the epithelial cells of the UC group, when compared to the CTR group (*p* < 0.05) (Figures 2(g) and 2(h)).

The ATF6 pathway was the last UPR branch evaluated. The ATF6*α* transcriptional levels showed no significant differences between the UC and control groups (*p* > 0.05), and a little variability of the transcriptional levels was verified among the patients (Figure 2(i)).

### 3.3. Chaperones and Genes Responsive to UPR Activation Are Upregulated in UC Patients

**Figure 3:**
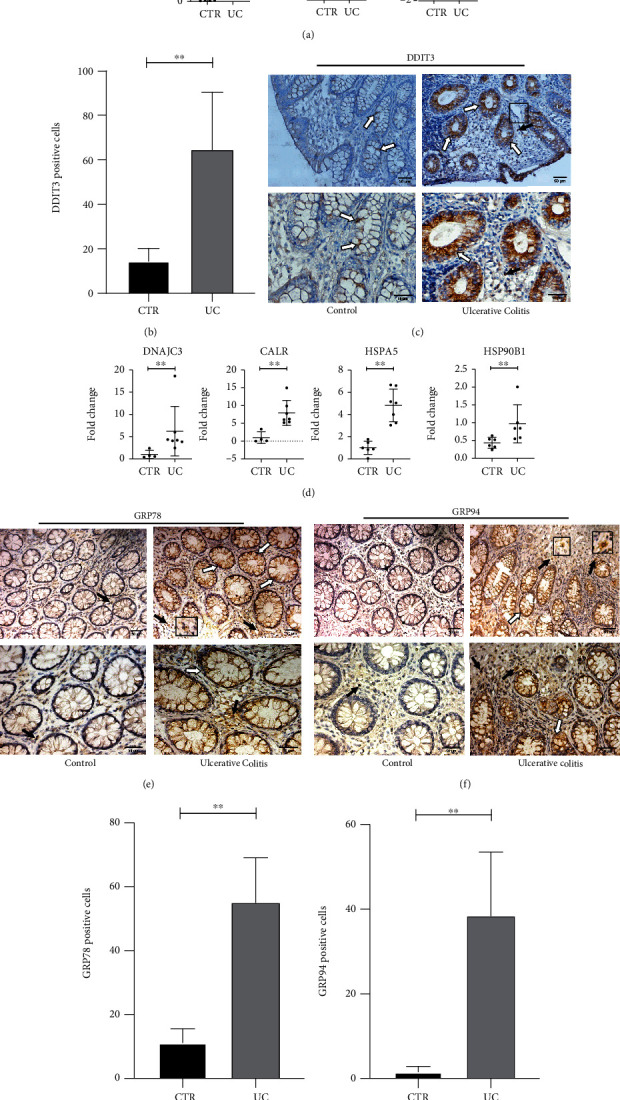
Activation of UPR-responsive genes and chaperone modulation. (a) Transcriptional levels of STC2, ATF3, and DDIT3 are shown as a fold change; ^∗^*p* < 0.05, ^∗∗^*p* < 0.005. (b) Quantitative analysis of immunohistochemistry staining for DDIT3 of both groups. For UC, *n* = 6; for CTR, *n* = 3; ^∗^*p* < 0.05. (c) Immunohistochemical analysis of DDIT3 was performed on paraffin-embedded slides from intestinal mucosa of both UC and CTR groups. The arrows indicate DDIT3-positive cells, which are shown in brown. The white arrows indicate positive epithelial cells, and the black arrows signalize positive cells from the lamina propria. Scale bars: 50 *μ*m. (d) Transcriptional levels of the chaperones DNAJC3, CALR, HSPA5, and HSP90B1 are shown as fold change, ^∗∗^*p* < 0.005. (e, f) Immunohistochemical analysis of GRP78 and GRP94 was performed on paraffin-embedded slides from intestinal mucosa of both groups. The arrows indicate GRP78- and GRP94-positive cells, which are shown in brown. The white arrows indicate positive epithelial cells, and the black arrows signalize positive cells from the lamina propria. Scale bars: 50 *μ*m. (g, h) Quantitative analysis of immunohistochemistry staining for GRP78 and GRP94 of both groups. (g) For UC, *n* = 8; for CTR, *n* = 5; ^∗∗^*p* < 0.005. (h) For UC, *n* = 6; for CTR, *n* = 4; ^∗∗^*p* < 0.005. CTR = control; UC = ulcerative colitis.

The regulation of UPR-related genes is triggered by ER stress activation. Therefore, we performed a transcriptional analysis of those genes in samples of colonic mucosa from UC and control patients. We observed a significant upregulation of calcium-regulatory protein stanniocalcin-2 (STC2) (*p* < 0.005), activating transcription factor-3 (ATF3) (*p* < 0.05), and DDIT3 (*p* < 0.05) (Figure 3(a)). In addition, we observed marked immunoreactivity for DDIT3 protein in the colonic epithelial cells of UC patients (Figures 3(b) and 3(c)), corroborating the DDIT3 transcriptional findings.

UPR activation can also increase the production of chaperones to resolve the accumulation of unfolded proteins in the ER. Therefore, we evaluated, through rt-PCR and immunohistochemistry techniques, whether the production of chaperones is modulated in intestinal biopsies from UC patients. Transcriptional analysis showed that UC patients presented a significant increase (*p* < 0.005) in the transcriptional levels of DnaJ heat shock protein family (DNAJC3) that encodes the DNAJC3 cochaperone, CALR that encodes the chaperone calreticulin, HSPA5 that encodes GRP78/BiP protein, and HSP90B1 that encodes the chaperone GRP94 (Figure 3(d)). In addition, the lamina propria and epithelial cells of UC intestinal mucosa showed a significantly higher immunoreactivity to GRP78 (Figures 3(e) and 3(g)) and GRP94 (Figures 3(f) and 3(h)) compared to the control group, confirming the transcriptional results.

### 3.4. Transcriptional Analysis of Apoptosis and Autophagy-Related Genes in UC Patients

**Figure 4:**
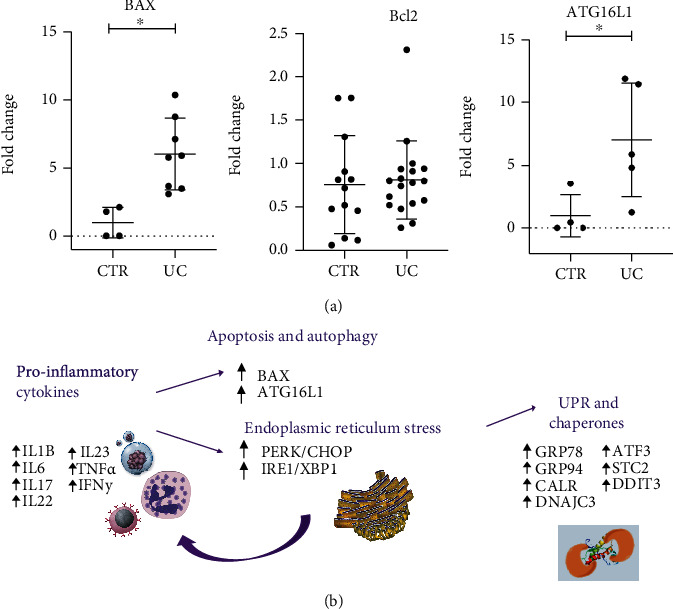
Apoptosis and autophagy are modulated in UC patients. (a) Transcriptional levels of BAX, Bcl-2, and ATG16L1 as determined by qPCR are shown as a fold change, ^∗^*p* < 0.05. UC = ulcerative colitis; CTR = control. (b) Illustrated chart of our results showing alterations in the pathways involving inflammation, ER stress, autophagy, and apoptosis markers in UC patients.

As apoptosis may occur as a result of inflammation and/or ER stress activation, we also evaluated the transcriptional levels of pro- and antiapoptotic genes. BAX was significantly upregulated in UC patients compared to the controls (*p* < 0.05), whereas Bcl-2 did not show modulation between the groups (Figure 4(a)). In addition, we evaluated whether there is autophagy modulation in biopsies from UC patients. rt-PCR analysis showed that ATG6L1 was significantly higher in UC patients compared to the CTR group (*p* < 0.05) (Figure 4(a)). These findings reinforce that ER stress is activated in UC patients, and mechanisms such as autophagy and apoptosis are attempts to counterbalance the tissue damage.  Figure 4(b) illustrates our findings.

## 4. Discussion

ER stress activation and UPR have been linked to inflammatory pathways in several diseases, such as autoimmune and infectious disorders [19]. Endoplasmic reticulum (ER) is an important organelle responsible for intestinal homeostasis and its stress and the UPR is essential to protect intestinal cells from infections, for example. In an experimental model (*AA^IEC^* mice), which is not capable to phosphorylate eIF2*α* (PERK pathway), Cao et al. [20] demonstrated an increased susceptibility to *Salmonella* Typhimurium infection and also to dextran sulfate sodium-induced colitis. Concerning IBDs, although the molecular mechanism that triggers ER stress activation in these affections is not completely understood, several studies have been reporting ER stress as one relevant component in the maintenance of the disease [17, 18, 21]. Most of the experimental studies using enteroids and colitis-induced animals have been suggesting that intestinal epithelial cells (IECs) are the main cell type where ER stress is activated [21–24]. Our results point towards this finding and our in situ hybridization and immunohistochemistry images also mainly showed ER stress markers in the IECs, besides cells from the lamina propria.

There are only a few studies that evaluated ER stress in UC using samples from colonoscopy examination. Tréton et al. [25] reported that the IRE1 and ATF6 but not PERK pathways were activated in UC patients. These different findings compared to our results may be due to the fact that Tréton et al. just analyzed noninflamed intestinal mucosa, and the occurrence of the inflammation in active UC itself may activate and exacerbate the ER stress, which leads in turn to the exacerbation of inflammatory pathways through NF-KB activation. The PERK pathway activates the expression of transcription factor ATF4, a DDIT3 inducer [26]. In our study, we observed that the PERK pathway is activated in the UC group. This activation contributed to the increased expression of DDIT3 in the IECs from UC patients. Lin et al. [27] showed that an extended PERK activity contributes to the induction of cell death. These results together with our own suggest that these cells activate apoptosis in order to restore homeostasis. In addition to the DDIT3 pathway, we observed increased BAX transcription, a proapoptotic gene, in UC patients.

Although we did not find a transcriptional modulation of ERN by qPCR, in situ hybridization and immunohistochemistry images showed the presence of ERN1 mRNA and sXbp-1, respectively, in intestinal samples of UC patients. When the UPR is initiated, all three pathways are activated at once. However, the duration of activation varies over time. Lin and colleagues [28] have confirmed, in an *in vitro* study, that IRE1 and ATF6 responses attenuate, whereas PERK activation remains, when the stress condition persists. Even though the exact moment of the disease evolution when this shift in UPR modulation takes place is unclear, UC patients included in our study, besides presenting disease activity at the time of inclusion, had an extended disease duration time (>10 years), characterized by chronic inflammation. Concerning the evaluation of ERN transcriptional levels, it was based on a previous experimental study that analyzed the activation of IRE1*α* (ERN1) in intestinal epithelial cells and not of IRE1*β* (ERN2). This activation leads to spontaneous colitis accompanied by loss of goblet cells and dysregulated epithelial barrier function [16]. The role of IRE1*α* is well established in the literature: when it is activated, it can bind to and activate the TNF*α* receptor-associated factor 2 in the cytoplasm, which activates NF-*κ*B, thus participating in inflammatory response and proapoptotic signalling in response to ER stress [29, 30]. In addition, IRE1*α* can interact with the proapoptotic BCL-2 family proteins (either BAX or BAK), thus contributing to apoptotic cell death [31]. Moreover, IRE1*α* may cause degradation of selective microRNA that usually represses translation of caspase-2, causing mitochondrial apoptosis [32].

The absence of transcriptional modulation of ATF6, the overproduction of cytokines, and the long period of disease activity altogether suggest that this pathway may be attenuated whereas PERK and IRE1 persist as a cell fate driving apoptosis. In addition, most of the consequence of ATF6 activation is related to cell adaptive response in order to restore ER homeostasis, including, among others, protein folding and/or protein degradation (via ERAD) [33]. We observed no significant differences in ATF6*α* transcriptional levels between UC and control groups, besides a little variability of this transcript among the patients, suggesting that this pathway may actually not be activated. On the other hand, our results suggest apoptosis pathway activation, but, under the conditions of our study, it may not be a consequence of ATF6*α* pathway activation, but rather a result of activation of other ER stress branches (CHOP/DDIT3).

Both ER stress activation and attempts of stress resolution are mediated by glucose-regulated protein (GRP-) 78 and 94 that work as chaperones, helping the ER restore balance [34]. Under physiological conditions, all three inactive ER stress pathways are linked to GRP78, also called binding immunoglobulin protein (BiP). Upon the accumulation of unfolded protein, GRP78 dissociates from the luminal domains of all three ER stress pathways, activating the UPR [34]. During UPR, GRP78 is also helpful in driving the unfolded proteins to degradation via ERAD [35]. Although the role of GRP94 is best known as an ER stress-related immune response mediator [36], it also assists the protein folding and targets misfolded protein to the ERAD [37]. In our study, we evaluated whether those UPR mediators were activated and showed that both HSP90B1 and HSPA5 are transcriptionally upregulated and expressed by both the epithelial and lamina propria cells in the UC group. UPR initiation induces the production of inflammatory genes via NF-*κ*B activation [38]. In fact, we observed high transcriptional levels of proinflammatory cytokines in UC colonic samples. These results support the characteristic proinflammatory profile seen in UC patients [39]. We also observed that IL17 and IL22 transcripts are upregulated in UC patients. This corroborates a recent study published by Powel and colleagues that showed that IL17 and IL22 promote ER stress and lead IECs to apoptosis in vitro. The authors also showed that the blockade of these cytokines in vivo reduces ER response in colonic epithelial cells [40].

One of the major protein degradation systems is the autophagy process, which is activated in order to maintain cellular homeostasis after a stress condition [41]. A genome-wide association study (GWAS) performed in 2006 identified the ATG16L1 as an autophagy-related gene for susceptibility to develop CD [42]. Since then, studies have reported that ATG6L1 is associated to CD, but not UC [43]. We observed, by rt-PCR, a significant increase in the ATG6L1 expression in the UC group, suggesting that there is no autophagy deficiency in UC patients. This result corroborates several other that have suggested that ER stress activation induces autophagy initiation [44–47].

It is worthwhile to consider some limitations of the study: we only evaluated patients with long duration of the disease, which may differ from recently diagnosed patients in the pattern of ER stress activation in the colonic mucosa. Moreover, patients were receiving different classes of drugs (immunosuppressors and biologics), which may influence the cytokine pattern and different ER stress-activated pathways. It is an observational study, and certain variables are not possible to control in order to have an adequate sample size. In spite of these limitations, our translational study brings new data on the predominance of the ER stress pathways in UC which contribute to a better understanding of this chronic disease.

## 5. Conclusions

We identified that two of the three ER stress pathways (PERK and IRE1) were activated in the colonic mucosa of UC patients, which may contribute to the maintenance of the inflammation in this tissue. UPR together with increased markers of apoptosis and autophagy reinforce that ER stress is activated, but they are not sufficient to control the inflammation and tissue damage in UC patients. These findings envision new molecular targets with potential therapeutic implications in the future. These could include strategies to block ER stress or promote the activity of the UPR pathways.

## Figures and Tables

**Table 1:** Demographic and clinical data from all patients included in the study.

	CTR	UC
Number of patients	17	22
Gender (masculine/feminine)	7/11	5/17
Age (median–min/max)	56 years old (35–69)	42 years old (27–75)
Time of UC (median–min/max)	—	11 years [1–25]
Disease extension (proctitis/left-side colitis/extensive colitis)	—	1/6/15
CRP (median–min/max)	—	0.6 (0.1–11)
Global Mayo score (median–min/max)	—	8.5 (1–11)
Endoscopic Mayo score (1/2/3/4)	—	0/5/3/14

Categorical variables are described as absolute frequencies and numerical variables as median (min–max). CTR = subjects without IBD; UC = patients with ulcerative colitis; CRP = C-reactive protein.

## Data Availability

All relevant data supporting the findings of this study are available within the paper.

## Supplementary Materials

Supplementary Materials Supplementary Figure: immune cell infiltrate of the lamina propria in the colonic mucosa of UC patients. (A) Representative hematoxylin and eosin (H&E) staining of colonic mucosa of ulcerative colitis (UC) and control (CTR) patients. Scale bars: 50 *μ*m. (B) Immune cell number of the lamina propria in the UC and CTR groups. For UC, *n* = 6; for CTR, *n* = 5; ^∗∗^*p* < 0.005.
