# Supplementary figures and images for: Endoplasmic Reticulum Stress in Colonic Mucosa of Ulcerative Colitis Patients Is Mediated by PERK and IRE1 Pathway Activation
Source: Mediators Inflamm. 2022 Feb 9;2022:6049500. doi: 10.1155/2022/6049500 (PMC8849912; doi:10.1155/2022/6049500)

**A**

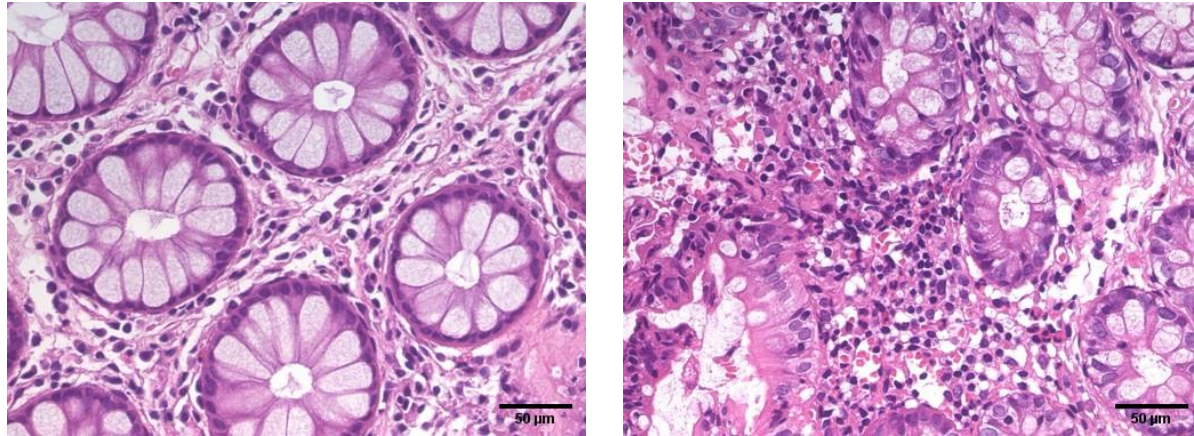

**B**

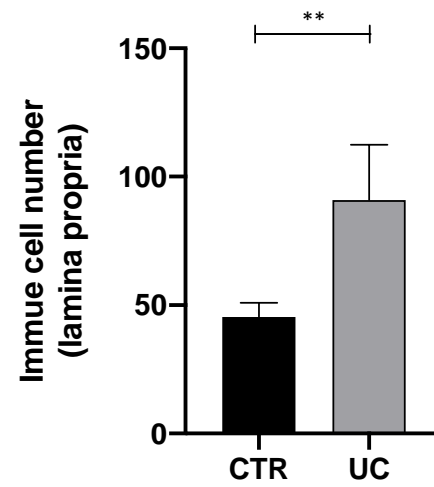

Supplement: Supplementary Materials — Supplementary Figure: immune cell infiltrate of the lamina propria in the colonic mucosa of UC patients. (A) Representative hematoxylin and eosin (H&E) staining of colonic mucosa of ulcerative colitis (UC) and control (CTR) patients. Scale bars: 50 μm. (B) Immune cell number of the lamina propria in the UC and CTR groups. For UC, n = 6; for CTR, n = 5; ∗∗p < 0.005. [file 6049500.f1.pdf]
